# Voice of People with Fragile X Syndrome and Their Families: Reports from a Survey on Treatment Priorities

**DOI:** 10.3390/brainsci9020018

**Published:** 2019-01-23

**Authors:** Jayne Dixon Weber, Elizabeth Smith, Elizabeth Berry-Kravis, Diego Cadavid, David Hessl, Craig Erickson

**Affiliations:** 1 National Fragile X Foundation, McLean, VA 22102, USA; 2 Cincinnati Children’s Hospital Medical Center Division of Child & Adolescent Psychiatry, Cincinnati, OH 45229, USA; 3 Departments of Pediatrics, Neurological Sciences, Biochemistry, Rush University Medical Center, Chicago, IL 60612, USA; Elizabeth_Berry-Kravis@rush.edu; 4 Fulcrum Therapeutics, Cambridge, MA 02139, USA; dcadavid@fulcrumtx.com; 5 MIND Institute and Department of Psychiatry and Behavioral Sciences, University of California Davis School of Medicine, Sacramento, CA 95817, USA; drhessl@ucdavis.edu; 6 Cincinnati Children’s Hospital Medical Center, Division of Child & Adolescent Psychiatry and the University of Cincinnati College of Medicine Department of Psychiatry and Behavioral Neuroscience, Cincinnati, OH 45229, USA; craig.erickson@cchmc.org

**Keywords:** fragile X syndrome, *FMR1* gene, voice of the person, voice of the patient, characteristics that have the greatest impact, developmental disorders

## Abstract

To date, there has been limited research on the primary concerns and treatment priorities for individuals with fragile X syndrome (FXS) and their families. The National Fragile X Foundation in collaboration with clinical investigators from industry and academia constructed a survey to investigate the main symptoms, daily living challenges, family impact, and treatment priorities for individuals with FXS and their families, which was then distributed to a large mailing list. The survey included both structured questions focused on ranking difficulties as well as qualitative analysis of open-ended questions. It was completed by 467 participants, including 439 family members or caretakers (family members/caretakers) of someone with FXS, 20 professionals who work with a person with FXS, and 8 individuals with FXS. Respondents indicated three main general areas of concern: Anxiety, behavioral problems, and learning difficulties. Important differences were noted, based on the sex and age of the individual with FXS. The results highlight the top priorities for treatment development for family members/caretakers, as well as a small group of professionals, and an even smaller group of individuals with FXS, while demonstrating challenges with “voice of the patient” research in FXS.

## 1. Introduction

Fragile X syndrome (FXS) is a neurodevelopmental condition that is caused by the expansion of the CGG repeat in the 5′ untranslated region of fragile X mental retardation 1 (*FMR1*) gene located on the X chromosome [1]. This expansion leads to methylation of the *FMR1* promoter, transcriptional silencing of the gene, and subsequent reduction or absence of fragile X mental retardation protein (FMRP). FMRP is an RNA-binding protein that regulates dendritic translation of many key synaptic proteins that influence synaptic function and plasticity [2]. FXS is the leading known inherited cause of intellectual disability. Individuals with FXS are most commonly diagnosed after presenting with language delay, and the majority of males with FXS will ultimately meet criteria for mild-to-severe intellectual disability [3]. The average IQ in men with FXS is 40–50, with a mental age of about of 5–6 years. Females with FXS are often less affected (average IQ about 80) than males, with about 25% having cognitive impairment and others frequently being diagnosed with learning disabilities [4]. There is a relatively consistent pattern of intellectual weaknesses (visuospatial skills, working memory, processing of sequential information, attention) and strengths (simultaneous processing, imitation, visual memory) characteristic of both males and females with FXS [2]. Multiple studies have shown a decrease in full-scale IQ scores with age as children with FXS become older [5,6]. Likewise, standard scores on the Vineland Adaptive Behavior Scale for overall adaptive behavior as well as subdomains have also been shown to decline with age during childhood, in males more so than in females with FXS [7]. Decline in standard scores for intelligence and adaptive function is not the result of loss of skills or regression but rather failure to keep pace with the normal rate of intellectual development. FXS is also associated with a constellation of behavioral symptoms, which can be highly problematic for functioning and family burden, including but not limited to high levels of anxiety, attention deficit/hyperactivity disorder, social communication deficits, and self-injurious and sensory-seeking behaviors [8].

Due to its known genetic and molecular underpinnings, symptoms of FXS can potentially be targeted via medical interventions. These interventions can focus on increasing expression of the missing FMRP protein and/or remediating downstream effects of neural and synaptic dysmaturation. End goals of treatment include both reducing symptom severity and improving activities of daily living (ADLs) and quality of life in affected individuals and their families. Given the array of behavioral and developmental symptoms that can exist in individuals with FXS, as well as the range of potential biochemical targets, it is useful to focus clinical research on symptoms and concerns that are experienced as most impairing to individuals with FXS and their families. Taking a patient-first perspective can be facilitated through qualitative research methods, where semistructured and free responses from patients and their families are used as primary data.

To date, there have been qualitative studies in FXS on topics including but not limited to diagnosis [9,10], communication impairments [11], physician knowledge [12], and technology use [13]. Closely related to the present work, Bailey and colleagues surveyed parents of children with FXS regarding the prevalence of developmental delay and eight other symptoms frequently associated with FXS. These included attention problems, hyperactivity, aggressive behaviors, self-injury, autism, seizures, anxiety, and depression [14]. Following this work, the same group [15] evaluated caregiver preferences for six different treatment foci (i.e., learning and applying new skills, explaining needs, controlling behavior, taking part in new social activities, caring for oneself, and paying attention). The highest priorities for treatment based on 614 responses from caregivers of males ages 5 years and older with FXS were (1) controlling behavior and (2) caring for oneself. These priorities were consistent across age groups. These results have been informative in directing treatment targets, but the present study has several features that can expand understanding of the needs of individuals with FXS and their families. These features include the addition of individuals with FXS as well as professionals to the survey, inclusion of females with FXS, inclusion of family members/caretakers of children under 5 years of age, and free response followed by coding, allowing participants to express interest in a wider range of treatment foci.

Here, we attempted to address these gaps and report on both quantitative and qualitative findings from an online survey completed by 467 respondents, including mostly family members/caretakers but also a small sample of professionals and individuals with FXS. The survey was designed around key problem areas (i.e., “concepts of interest”) as well as priorities for treatment. Survey items focused on major concerns, symptom areas, daily living skills, family impact, and treatment priorities, and results are reported by age and sex of the individual affected by FXS. A free-response item was also included to allow family members/caretakers to detail concerns above and beyond those offered, as a goal of this study was to use a patient-first approach without a priori hypotheses regarding expected reporting patterns for FXS family members/caretakers and professionals.

## 2. Methods

Survey: Initial survey questions were created within a focus group of individuals with expertise in FXS via their involvement in the National Fragile X Foundation. The initial draft survey included both structured, forced-choice questions as well as open-ended questions, with questions focused on the following information: (1) Respondent characteristics, including individuals with FXS; (2) major concerns and symptoms experienced; (3) difficulties with daily living skills; (4) family impact; and (5) treatment priorities. The survey was divided into age groups based on standard developmental stages: Early childhood, middle childhood, adolescent/young adult, and adult. This draft survey was then presented to a focus group including parents of individuals with FXS and medical providers, with the purpose of confirming that the survey was comprehensive, clear, and respectfully worded. Minor revisions were made, incorporating the focus group feedback. Subsequently, the final survey was sent via email to four families of individuals with FXS who had volunteered to pilot the process involved in completing the survey. No further adjustments were requested based on this pilot sample. No identifying information was included in the survey. The final survey can be seen in the Appendix A.

Sample: A link to the survey (via SurveyMonkey) was sent to 10,000+ emails subscribed to receive general emails from the National Fragile X Foundation. Recipients of the email were eligible to participate if they were an individual with a full FXS mutation or were a family member or caretaker of an individual with a full FXS mutation or a professional who works with an individual with a full FXS mutation. For individuals associated with multiple children with FXS, these individuals were eligible to complete the survey once per affected child.

Analyses: For items where responses required ranking concerns, we included data from participants who did not use all ranks (e.g., only assigning ranks of 1 and 2 and leaving all others blank) but excluded rank data higher than those instructed (e.g., assigning a rank of “6” when instructed to rank top 5). To compare ranked items, a weighted mean rank score was calculated as follows. First, the number 1 ranked item was given a 5, the number 2 ranked item was given a 4, and so on. Then, the sum was calculated for each item and then divided by the number of people who completed the survey. See the Supplementary Tables S1–S5.

For survey items concerning overall problems, problematic symptoms, daily living challenges, and family impact, we therefore report rates of endorsement as well as weighted mean rank scores across the sample and within groups by age and sex. For open-ended responses, data were first open coded for themes, and keyword lists were constructed for each theme. Then, all responses were coded in vivo for presence of any of the key words, with presence of a key word indicating endorsement of a theme.

## 3. Results

**Figure 1:**
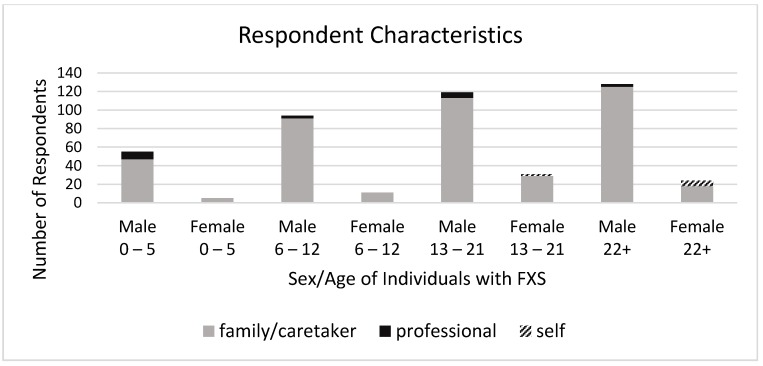
Survey respondent characteristics. FXS: Fragile X syndrome.

There were 467 individual responses to the survey, including 8 individuals with Fragile X (i.e., endorsing “I have Fragile X syndrome”), 20 professionals (i.e., endorsing “I am a professional who works with a person with FXS”), and 439 family members or caretakers (i.e., endorsing “I am a family member or caretaker of someone with FXS”). It should be noted that not everyone answered every question. Reporters described the individual with FXS on whom they were reporting as being mostly males (*n* = 397, 84.8%), with ages distributed across the lifespan (see Figure 1). See the data in Supplementary Table S1.

### 3.1. Results from Family Members/Caretakers

**Figure 2:**
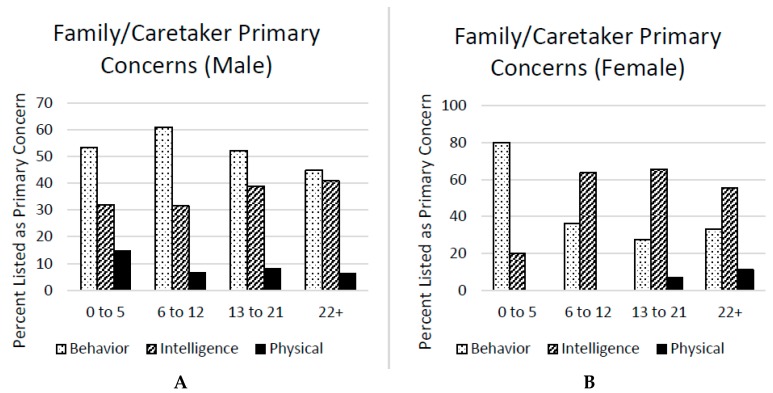
Primary concerns of family members/caretakers of males (**A**) and females (**B**) with FXS.

Major Concerns. Respondents completed the following question: “Rank these three areas (behavior, intelligence, physical abilities) from one to three to the extent it affects the person’s daily life–with one having the greatest impact and three having the least impact:” No other instructions or definitions were provided for this question on the survey. Each of the three areas of impact was described as having the greatest impact by at least some family members/caretakers, with behavior endorsed most commonly as having the greatest impact for males alone. However, for females alone, intelligence was endorsed as having the greatest impact. This question was answered by 429 family members/caretakers (see Figure 2). Physical abilities were the main concern in about 15% of FXS males aged 0–5 and 10% of FXS females age 22+ but were the main concern in less than 10% of all other groups. Behavior was by far the main concern in FXS males age 12 and under; however, in each older age category, intelligence was the main concern in a larger percent of males and, by age 22+, the main concern was divided almost equally between behavior and intelligence, suggesting that intellectual deficits are perceived as increasingly limiting as FXS males (and females) become older.

**Figure 3:**
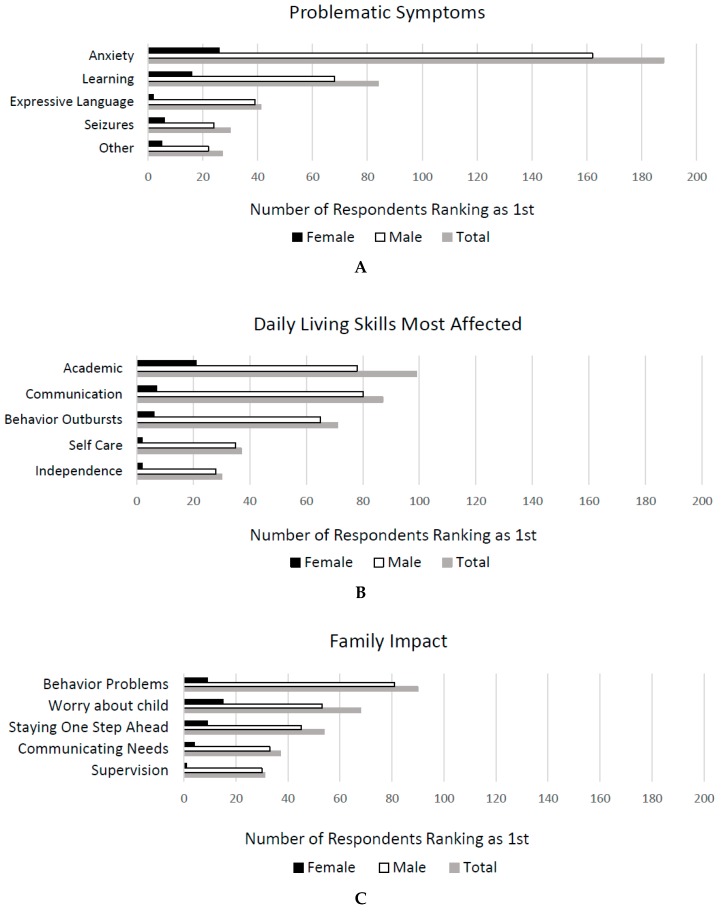
Family member/caretaker’s 1st rank for characteristics that have the greatest impact on the life of the person with FXS (**A**), daily living skills most affected (**B**), family impact (**C**).

Problematic Symptoms. Family members/caretakers (438) completed the following question: “Check the five characteristics that have the greatest impact on the life of the person with FXS. Prioritize 1, 2, 3, 4, 5.” Out of potential symptoms listed (please see Appendix A for a full list), the following 5 symptoms had the highest weighted mean score (see Figure 3A): (1) Anxiety–anticipatory, e.g., of new/upcoming events and or social anxiety; (2) learning or intellectual disability (problems with abstract thinking, learning); (3) speech/language delays–expressive (speaking spoken language); (4) seizures; and (5) other. Anxiety was rated highest in males beginning at age 6 and in females across all ages, although in females, anxiety was rated highest at similar rates to learning problems. In young males (ages 0–5), expressive language delays were described as being the most problematic symptom. See the data in Supplementary Table S2.

Daily Living Skills Most Affected. Family members/caretakers (436) answered the question “Check the top five areas of daily life the person with FXS is most affected by. Prioritize 1, 2, 3, 4, 5.” Out of 15 possible areas, the five abilities that received the highest weighted rank score were (see Figure 3B): (1) Ability to learn academic skills/reading/math; (2) ability to speak/communicate; (3) ability to control behavioral outbursts; (4) ability to take care of self; and (5) independence. Again, ability to speak was a higher-rated concern in the 0–5 group than the others; however, this was one of the most highly rated items throughout childhood and adolescence, while in adulthood, ability to live independently became the highest rated daily living skill concern. In general, ablity to speak, learn academics, control behavior, and perform self-care were fairly evenenly rated as the skills weighted as most problematic across all age groups of males, while highest rated daily living problems for females reflected more social issues. See the data in Supplementary Table S3.

Family Impact. Family members/caretakers (431) answered the question “Which five specific aspects of daily living with FXS are the most challenging? Prioritize 1, 2, 3, 4, 5.” Out of twenty potential options, the following five aspects were ranked as the most challenging: (1) Handling behaviors (negative)—tantrums, aggression, spitting, cussing; (2) worry about the future; (3) always thinking—how are things going, what do I need to do next? Needing to always be ‘one step’ ahead; (4) person is unable to tell you what he/she wants/needs; and (5) supervision (see Figure 3C). See the data in Supplementary Table S4.

**Figure 4:**
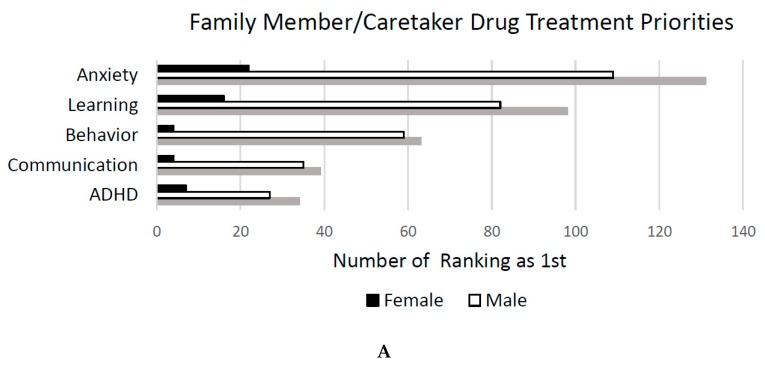

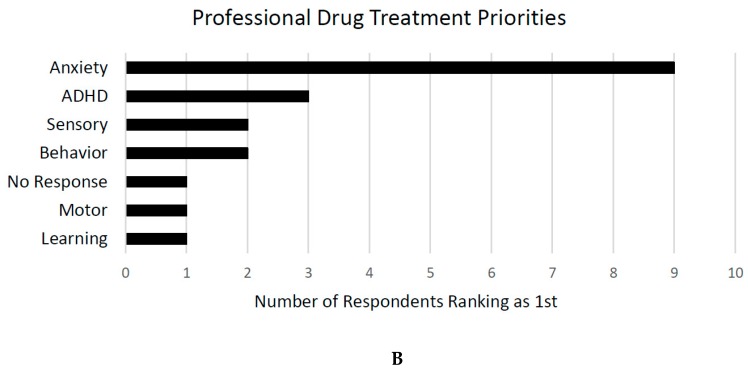
Family/caretaker (**A**) and professional drug (**B**) treatment priorities.

Treatment Priorities. Participants responded to the question “What are the top three aspects of Fragile X syndrome that you would like to see a drug treatment address, list in order of preference, with the most important one first.” After open coding of all three listed responses for all participants, the following themes emerged: Anxiety (e.g., “anxiety”, “social anxiety”, “reduce anxiety”), learning (e.g., “intellect”, “cognitive abilities”, “learning issues”), behaviors (e.g., “behavioral outbursts”, “tantrums”, “aggression”), Attention Deficit/Hyperactivity Disorder (ADHD) (e.g., “attention span”, “focus”, “impulse control”), communication (e.g., “speech delay”, “communication delay”, “language”), sensory (e.g., “sensory processing”, “hyperarousal”, “hand biting”), social (e.g., “social skills”, “social behaviors”, “connecting with others”), perseveration (“perseverative behaviors”, “saying the same thing”, “perseveration”), sleep (e.g., “sleep”, “sleeping”, “not sleeping”), mood (e.g., “depression”, “mood stability”, “mood swings”), motor (e.g., “fine motor skills”, “coordination”, “low muscle tone”), autism (e.g., “autism”, “autistic behavior”, “autistic tendencies”), eating (e.g., “weight”, “hunger”, “curb appetite”), and seizures (e.g., “seizures”, “seizure reduction”, “seizure control”). Notably, two family members/caretakers indicated that they were not interested in development of pharmaceutical treatments for symptoms of FXS. This question was answered by 439 family members/caretakers (see Figure 4A). See the data in Supplementary Table S5.

### 3.2. Voice of Professionals

Twenty professionals completed the survey. While this sample size prohibited investigations of mean response frequencies by age and sex for forced-choice questions, themes from free responses to the question about medication priorities are summarized below (see Figure 4B). Anxiety was most frequently described as the 1st priority for treatment, with almost half of professionals surveyed listing anxiety for their first priority.

### 3.3. Voice of Individuals with FXS

**Figure 5:**
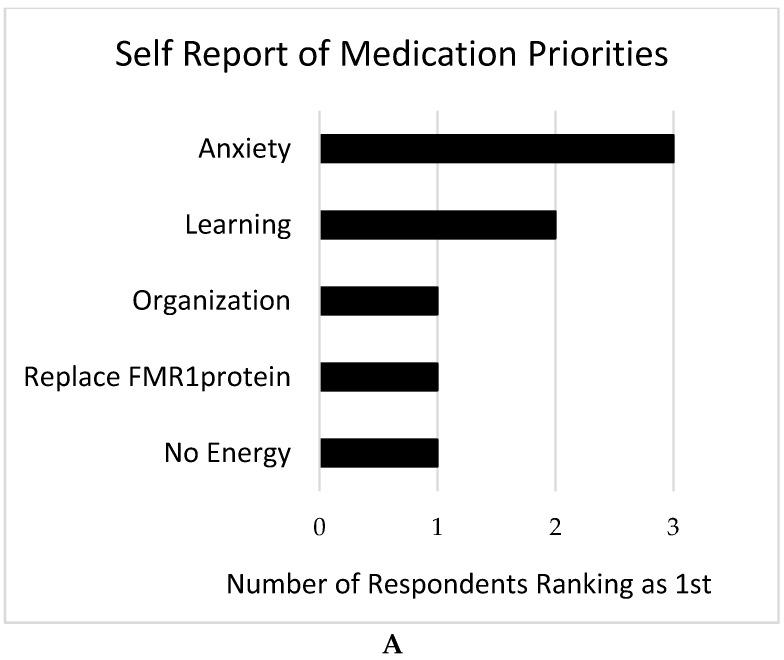

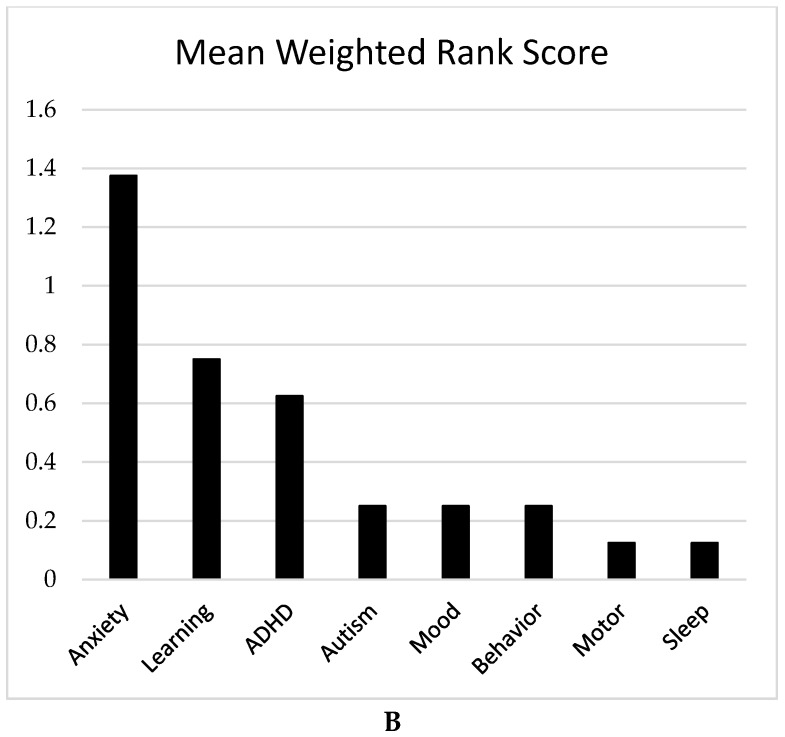
Drug treatment priorities as reported by 8 females with FXS. **A**: Drug treatment priorities ranked first as reported by 8 females with FXS. **B**: Drug treatment priorities, as a mean weighted rank score of all responses, as reported by 8 females with FXS.

Only 8 individuals with FXS completed this survey, all of whom were female and ages 13 years or older. Given this small number, we focus here on individual answers to the free response question rather than group means for the forced-choice questions. In parallel with responses from families, anxiety was most commonly listed as a priority for treatment for this group of individuals with FXS (see Figure 5A). Due to the limited number of respondents, a weighted mean score was calculated as described above for all of the responses received (see Figure 5B).

## 4. Discussion

This paper presents family member/caretaker/professional/self-reported information on the characteristics of FXS that have the greatest impact on the daily lives of people living with FXS and their families/professionals and the key areas of need for treatments. Responses highlight the role of anxiety as well as some other key symptoms in the lives of individuals with FXS and also demonstrate some of the challenges that can be encountered in “voice of the person” research within this population.

While one goal of this research was to obtain information directly from individuals with FXS, only eight of the respondents were individuals with FXS themselves (all female), with around 90% of responses coming from family members/caretakers. It is likely that this distribution was skewed toward females because females tend to have more typical cognitive abilities, making the survey more accessible to them. If males with FXS were able to communicate their concerns, based on severity differences alone, they would likely communicate different concerns than females with FXS, and in fact, parents of males reported a different pattern of primary concerns than those of females. Most individuals with FXS, males in particular, would need significant support to provide responses in the format presented, and for many, intellectual impairment would make the task prohibitive. Even females who are capable of carrying out a self-report task have been shown to be fairly inaccurate and erratic in their ratings. This may be due to deficits in understanding quantity, which would interfere with assignment of severity. While this is partially alleviated by the included free response questions, this format relies on significant receptive and expressive language skills. Females with FXS may also have difficulty identifying their own problems as well, or the degree to which they differ from experiences of typically developing females. Therefore, while future work could use other adaptive methods for qualitative research (e.g., transcription of patient’s statements as a way to reduce demand for reading and writing skills), integration of individual’s described experiences with those of family members/caretakers and professionals will be essential for a comprehensive understanding of the challenges faced by individuals with FXS.

Importantly, while survey responses varied, three main concerns emerged as consistently problematic in the lives of people with FXS: Learning/cognitive problems, anxiety, and behavior problems. The importance of each of these concerns varied depending on the focus of the question. Specifically, anxiety was described as the most problematic symptom of FXS, while behavior problems were listed as most difficult for family members/caretakers, and learning was the most consistently reported daily living problem. Anxiety was described as a top treatment priority by family members/caretakers, professionals, and individuals with FXS alike. Anxiety, however, as defined in DSM5, is a symptom that is perceived by the patient and depends on self-report. As defined this way, anxiety is very hard to assess in most patients with FXS, given the assessment is by proxy. As such, it will be important to understand the manifestations and symptoms observed by the family members/caretakers when they report an individual with FXS as having anxiety. These observable symptoms would be expected to include: Social avoidance, anticipation of upcoming events with repeated questioning and need for constant reassurance, difficulty with performance when directly requested and while being observed, inability to transition, approach–withdrawal behaviors, eye aversion, and signs of “fight or flight” when the individual is stressed, followed by aggressive outbursts. Further qualitative work should be done to elucidate and document the types and frequencies of behaviors observed and reported as a correlate to anxiety experienced in the daily lives of individuals with FXS, as these symptoms can vary widely across the phenotype.

This study is an important first step in establishing a stakeholder-first perspective on priorities for treatment in FXS. However, several limitations can be noted. Due to its online nature, all of the information was self-reported, and we could neither confirm an FXS diagnosis nor determine the functioning level of the individuals with FXS. Many options were offered in order to capture potential areas of concern, but as a result, some participants may have found the choices confusing. In addition, most respondents were family members or caretakers, and this limits our ability to understand treatment priorities for individuals with FXS themselves. While a sample of females with FXS completed this study, that sample was relatively small and likely not representative of all individuals with FXS. Future studies directed more specifically at characterizing treatment priorities in a larger group of females with FXS are needed and should be performed. Future studies would also benefit from a design that allows individuals with FXS to self-report by including response formats and wording of questions that are more accessible to those with intellectual disability. However, due to the nature of FXS, integration with family members/caretakers and professional reports will be essential for understanding the patient perspective.

The clinical implications of these survey findings include continued focus by clinicians and researchers on the three primary concerns noted by survey respondents. Specifically, while participants noted many areas of concern and difficulty, symptoms related to learning, anxiety, and behavior problems are reported to cause the most difficulty for individuals with FXS and their families. There was, however, significant variability in response patterns across age and sex, and these reported differences will dictate a focus on different clinical features and different clinical outcome assessments for therapeutic trials, depending on the FXS age/gender subgroup being targeted in the trial.

## Supplementary Materials

The following are available online at http://www.mdpi.com/2076-3425/9/2/18/s1, Table S1: Percent ranked first and weighted rank score by sex and age group for this question: Rank these three areas from one to three to the extent it affects the person’s daily life—with one having the greatest impact and three having the least impact. (Primary Concerns), Table S2: Percent ranked first and weighted rank score by sex and age group for Question: Check the five characteristics that have the greatest impact on the life of the person with FXS. Prioritize 1, 2, 3, 4, 5. (Problematic Symptoms), Table S3: Percent ranked first and weighted rank score by sex and age group for this question: Check the top five areas of daily life the person with FXS is most affected by. Prioritize 1, 2, 3, 4, 5. (Daily Living Skills Most Affected), Table S4: Percent ranked first and weighted rank score by sex and age group for this question: Which five specific aspects of daily living with FXS are the most challenging? Prioritize 1, 2, 3, 4, 5 (Family Impact), Table S5: Percent ranked first and weighted rank score by sex and age group for this question: What are the top three aspects of Fragile X syndrome that you would like to see a drug treatment address, list in order of preference, with the most important one first. (Drug Treatment Priorities).

## Appendix A

Voice of people with Fragile X syndrome and their families: Reports from a survey on treatment priorities

1. Which of the following best describes you?
  __ I have Fragile X syndrome
  __ I am a family member or caretaker of someone with FXS
  __ I am a professional who works with a person with FXS
2. What is the age of the person with FXS with whom you have the connection? Or your age if you have FXS.
  __ Male: Birth to 5 years old
  __ Female: Birth to 5 years old
  __ Male: 6 to 12 years old
  __ Female: 6 to 12 years old
  __ Male: 13 to 21 years old
  __ Female: 13 to 21 years old
  __ Male: 22 years and older
  __ Female: 22 years and older
3. Check the five characteristics that have the greatest impact on the life of the person with FXS. Prioritize 1, 2, 3, 4, 5.
  __ Anxiety—anticipatory, e.g., of new/upcoming events
  __ Auditory processing difficulties—being able to listen to instructions and react to them
  __ Autism
  __ Communication delays—initiation (asking for help) and social (turn-taking)
  __ Hyperactivity
  __ Learning or Intellectual disability (problems with abstract thinking, learning)
  __ Memory—short term
  __ Memory—long term
  __ Motor delays (e.g., low muscle tone, poor fine motor skills, poor balance)
  __ Motor stereotypes (hand-flapping, spinning around)
  __Perseveration—speech (repeating things over and over)
  __ Seizures
  __ Sensory processing difficulties
  __ Short attention span
  __ Social anxiety
  __ Speech/Language delays—receptive (understanding spoken language)
  __ Speech/Language delays—expressive (speaking spoken language)
  __ Visual information processing difficulties
  __ Other - Describe
4. Rank these three areas from one to three to the extent it affects the person’s daily life—with one having the greatest impact and three having the least impact:
  __ Behavior
  __ Intelligence
  __ Physical abilities
5. Check the top five areas of daily life the person with FXS is most affected by. Prioritize 1, 2, 3, 4, 5.
  __ Ability to learn academic skills/reading/math
  __ Ability to take care of self-care skills/hygiene/cooking
  __ Ability to speak/communicate
  __ Ability to be left alone/spend time alone
  __ Ability to control behavioral outbursts
  __ Ability to attend and perform at school
  __ Ability to find/maintain job
  __ Ability to make and maintain friends
  __ Ability to live independently
  __ Ability to establish and maintain a relationship
  __ Ability to be like other people his/her age.
  __ Ability to attend events where there are a lot of people/noise
  __ Willingness to go to new places
  __ Willingness to travel/ go on vacation
  __ Other—Describe
6. Which five specific aspects of daily living with FXS are the most challenging? Prioritize 1, 2, 3, 4, 5.
  __ Always thinking—how are things going, what do I need to do next? Needing to always be ‘one step’ ahead
  __ Checking in/setting up daily programming—school, work, etc.
  __ Doctor/dentist appointments—finding/attending
  __ Doing activities with friends—both child’s and adult’s
  __ Extra costs—therapies, medications, clothing, glasses, laundry
  __ Extra time it takes to do everything
  __ Finding respite
  __ Food—Always hungry/wants to eat out
  __ Handling behaviors (negative)—tantrums, aggression, spitting, cussing
  __ Hygiene—shower, toileting, hair cuts
  __ Impact on non-affected family members
  __ Medications—getting prescriptions/not running out/ making changes
  __ Need for constant supervision
  __ Needing to make sure everything is “set” for the day—routine, visuals
  __ Person doesn’t understand directions/can only do one thing at a time
  __ Person is unable to tell you what he/she wants/needs
  __ Running errands—how many stops can I make? What environments could be hard/noisy?
  __ Sleeping
  __ Worry about the future
  __ Other—Describe
7. List three of your favorite things about the person with FXS. (Or three things you like about yourself).
8. What are the top three aspects of Fragile X syndrome that you would like to see a drug treatment address, list in order of preference, with the most important one first.

## References

[1] Verkerk A.J., Pieretti M., Sutcliffe J.S., Fu Y.H., Kuhl D.P., Pizzuti A., Reiner O., Richards S., Victoria M.F., Zhang F.P. (1991). Identification of a gene (*FMR-1*) containing a CGG repeat coincident with a breakpoint cluster region exhibiting length variation in fragile X syndrome. Cell.

[2] Gross C., Hoffmann A., Bassell G.J., Berry-Kravis E.M. (2015). Therapeutic Strategies in Fragile X Syndrome: From Bench to Bedside and Back. Neurotherapeutics.

[3] Kaufmann W.E., Abrams M.T., Chen W., Reiss A.L. (1999). Genotype, molecular phenotype, and cognitive phenotype: Correlations in fragile X syndrome. Am. J. Med. Genet..

[4] De Vries B.B., Wiegers A.M., Smits A.P., Mohkamsing S., Duivenvoorden H.J., Fryns J.P., Curfs L.M., Halley D.J., Oostra B.A., van den Ouweland A.M. (1996). Mental status of females with an *FMR1* gene full mutation. Am. J. Med. Genet..

[5] Fisch G.S., Carpenter N., Holden J.J., Howard-Peebles P.N., Maddalena A., Borghgraef M., Steyaert J., Fryns J.P. (1999). Longitudinal changes in cognitive and adaptive behavior in fragile X females: A prospective multicenter analysis. Am. J. Med. Genet..

[6] Fisch G.S., Simensen R., Tarleton J., Chalifoux M., Holden J.J.A., Carpenter N., Howard-Peebles P.N., Maddalena A. (1996). Longitudinal study of cognitive abilities and adaptive behavior levels in fragile X males: A prospective multicenter analysis. Am. J. Med. Genet..

[7] Klaiman C., Quintin E.-M., Jo B., Lightbody A.A., Hazlett H.C., Piven J., Hall S.S., Chromik L.C., Reiss A.L. (2014). Longitudinal profiles of adaptive behavior in fragile X syndrome. Pediatrics.

[8] Hagerman R.J., Berry-Kravis E., Kaufmann W.E., Ono M.Y., Tartaglia N., Lachiewicz A., Kronk R., Delahunty C., Hessl D., Visootsak J. (2009). Advances in the treatment of fragile X syndrome. Pediatrics.

[9] Visootsak J., Charen K., Rohr J., Allen E., Sherman S. (2012). Diagnosis of fragile X syndrome: A qualitative study of African American families. J. Genet. Couns..

[10] Anido A., Carlson L.M., Taft L., Sherman S.L. (2005). Women’s attitudes toward testing for fragile X carrier status: A qualitative analysis. J. Genet. Couns..

[11] Brady N., Skinner D., Roberts J., Hennon E. (2006). Communication in young children with fragile X syndrome: A qualitative study of mothers’ perspectives. Am. J. Speech-Lang. Pathol..

[12] Budimirovic D.B., Cvjetkovic S., Bukumiric Z., Duy P.Q., Protic D. (2018). Fragile X-Associated Disorders in Serbia: Baseline Quantitative and Qualitative Survey of Knowledge, Attitudes and Practices Among Medical Professionals. Front. Neurosci..

[13] Raspa M., Fitzgerald T., Furberg R.D., Wylie A., Moultrie R., DeRamus M., Wheeler A.C., McCormack L. (2018). Mobile technology use and skills among individuals with fragile X syndrome: Implications for healthcare decision making. J. Intellect. Disabil. Res.: JIDR.

[14] Bailey D.B., Raspa M., Olmsted M., Holiday D.B. (2008). Co-occurring conditions associated with *FMR1* gene variations: Findings from a national parent survey. Am. J. Med. Genet. Part A.

[15] Cross J., Yang J.C., Johnson F.R., Quiroz J., Dunn J., Raspa M., Bailey D.B. (2016). Caregiver Preferences for the Treatment of Males with Fragile X Syndrome. J. Dev. Behav. Pediatri.: JDBP.

