# Supplementary material for: Voice of People with Fragile X Syndrome and Their Families: Reports from a Survey on Treatment Priorities
Source: Brain Sci. 2019 Jan 23;9(2):18. doi: 10.3390/brainsci9020018 (PMC6406416; doi:10.3390/brainsci9020018)
Supplement: Supplementary file 1 [file brainsci-09-00018-s001.pdf]

Supplementary Tables

Table S1. Percent ranked first and weighted rank score by sex and age group for this question: Rank these three areas from one to three to the extent it affects the person's daily life—with one having the greatest impact and three having the least impact. (Primary Concerns).

Q1 Primary Concerns	Sex/Age Group		Percent Ranked "1st"	Weighted Rank Score
Behavior	Male	0–5	53.2	2.3
		6–12	60.9	2.5
		13–21	52.2	2.3
		22+	44.8	2.1
	Female	0–5	80	2.8
		6–12	36.4	2.3
		13–21	27.6	1.9
		22+	30	1.9
Intelligence	Male	0–5	31.9	2.2
		6–12	31.5	2.1
		13–21	38.9	2.2
		22+	40.8	2.1
	Female	0–5	20	2.2
		6–12	63.6	2.5
		13–21	65.5	2.6
		22+	55.6	2.3
Physical	Male	0–5	14.9	1.5
		6–12	6.5	1.3
		13–21	8.0	1.3
		22+	6.4	1.2
	Female	0–5	0	0.8
		6–12	0	1.2
		13–21	6.9	1.5
		22+	11.1	1.3

Shading indicates highest percent and highest weighted rank score per sex and age group.

Table S2. Percent ranked first and weighted rank score by sex and age group for Question: Check the five characteristics that have the greatest impact on the life of the person with FXS. Prioritize 1, 2, 3, 4, 5. (Problematic Symptoms).

Q2 Problematic Symptoms	Sex/Age Group		Percent Ranked 1st	Mean Weighted Rank Score
Anxiety	Male	0–5	17.0	1.43
		6–12	35.9	2.97
		13–21	32.7	2.73
		22+	43.2	3.04
	Female	0–5	36.4	0.4
		6–12	27.6	2.27
		13–21	27.8	2.52
		22+	14.3	2.61
Learning	Male	0–5	10.6	1.53
		6–12	20.6	2.53
		13–21	23.9	2.40
		22+	13.6	2.22
	Female	0–5	36.4	1.4
		6–12	24.1	2.82
		13–21	27.8	2.62
		22+	6.3	2.39
Expressive Language	Male	0–5	23.4	2.53
		6–12	14.1	1.29
		13–21	6.2	1.48
		22+	6.4	0.96
	Female	0–5	0	1.6
		6–12	3.4	0.55
		13–21	5.6	0.72
		22+	4.8	0.72
Seizures	Male	0–5	6.4	0.32
		6–12	7.6	0.48
		13–21	8.0	0.42
		22+	4	0.21
	Female	0–5	9.1	1
		6–12	10.3	0.45
		13–21	5.6	0.55
		22+	1.6	0.44
Other	Male	0–5	6.4	0.45
		6–12	5.4	0.36
		13–21	6.2	0.59
		22+	5.6	0.46
	Female	0–5	0	1
		6–12	13.8	0
		13–21	0	1.14
		22+	4.8	0.28

Shading indicates highest percent and highest weighted rank score per sex and age group.

Table S3. Percent ranked first and weighted rank score by sex and age group for this question: Check the top five areas of daily life the person with FXS is most affected by. Prioritize 1, 2, 3, 4, 5. (Daily Living Skills Most Affected).

Q3 Daily Living Skills Most Affected	Sex/Age Group		Percent Ranked 1st	Mean Weighted Rank Score
Academic	Male	0–5	12.77	1.77
		6–12	32.61	2.74
		13–21	26.55	2.50
		22+	9.60	1.46
	Female	0–5	0.00	3.40
		6–12	63.64	4.09
		13–21	27.59	2.34
		22+	33.33	2.00
Communication	Male	0–5	48.94	3.36
		6–12	16.30	1.97
		13–21	22.12	1.88
		22+	13.60	1.26
	Female	0–5	40.00	2.60
		6–12	9.09	1.27
		13–21	13.79	1.21
		22+	0.00	0.67
Behavior Outbursts	Male	0–5	19.15	2.49
		6–12	20.65	2.52
		13–21	13.27	1.58
		22+	17.60	1.51
	Female	0–5	20.00	2.40
		6–12	9.09	2.55
		13–21	10.34	1.10
		22+	5.56	0.72
Self Care	Male	0–5	6.38	1.47
		6–12	10.87	1.62
		13–21	9.73	1.98
		22+	8.80	1.57
	Female	0–5	0.00	2.40
		6–12	0.00	1.18
		13–21	3.45	0.66
		22+	5.56	0.94
Independence	Male	0–5	0.00	0.17
		6–12	0.00	0.18
		13–21	6.19	1.08
		22+	16.80	2.15
	Female	0–5	20.00	1.00
		6–12	0.00	0.09
		13–21	3.45	0.86
		22+	0.00	0.67

Shading indicates highest percent and highest weighted rank score per sex and age group.

Table S4. Percent ranked first and weighted rank score by sex and age group for this question: Which five specific aspects of daily living with FXS are the most challenging? Prioritize 1, 2, 3, 4, 5 (Family Impact).

Q4 Family Impact	Sex/Age Group		Percent Ranked 1st	Mean Weighted Rank Score
Behavior Problems	Male	0–5	29.79	2.36
		6–12	27.17	2.33
		13–21	19.47	1.74
		22+	16.00	1.39
	Female	0–5	40.00	2.40
		6–12	18.18	2.27
		13–21	10.34	0.79
		22+	11.11	1.06
Worry About Child	Male	0–5	6.38	1.23
		6–12	5.43	0.90
		13–21	19.47	1.88
		22+	18.40	1.95
	Female	0–5	0.00	1.80
		6–12	9.09	1.09
		13–21	34.48	2.34
		22+	22.22	1.94
Staying One Step Ahead	Male	0–5	4.26	0.98
		6–12	10.87	1.45
		13–21	16.81	1.40
		22+	11.20	1.30
	Female	0–5	0.00	0.00
		6–12	9.09	0.82
		13–21	20.69	2.21
		22+	11.11	1.06
Communicating Needs	Male	0–5	23.40	1.66
		6–12	5.43	0.88
		13–21	6.19	0.86
		22+	8.00	1.09
	Female	0–5	40.00	2.40
		6–12	0.00	0.64
		13–21	6.90	0.48
		22+	0.00	0.17
Supervision	Male	0–5	8.51	1.19
		6–12	9.78	1.42
		13–21	6.19	1.29
		22+	8.00	0.93
	Female	0–5	0.00	1.00
		6–12	0.00	0.82
		13–21	3.45	0.52
		22+	0.00	0.50

Shading indicates highest percent and highest weighted rank score per sex and age group.

Table S5. Percent ranked first and weighted rank score by sex and age group for this question: What are the top three aspects of Fragile X syndrome that you would like to see a drug treatment address, list in order of preference, with the most important one first. (Drug Treatment Priorities).

Q5 Drug Treatment Priorities	Sex/Age Group		Percent Ranked 1st	Mean Weighted Rank Score
Anxiety	Male	0–5	6.38	0.79
		6–12	25.00	1.32
		13–21	31.86	1.44
		22+	37.60	1.67
	Female	0–5	0.00	1.00
		6–12	27.27	1.00
		13–21	41.38	1.59
		22+	38.89	1.67
Learning	Male	0–5	23.40	0.96
		6–12	22.83	1.13
		13–21	23.89	1.16
		22+	18.40	0.86
	Female	0–5	40.00	1.20
		6–12	9.09	0.91
		13–21	31.03	1.55
		22+	22.22	0.89
Behavior	Male	0–5	12.77	0.60
		6–12	17.39	0.84
		13–21	16.81	0.79
		22+	14.40	0.78
	Female	0–5	0.00	0.00
		6–12	18.18	0.91
		13–21	6.90	0.38
		22+	0.00	0.06
Communication	Male	0–5	23.40	1.36
		6–12	6.52	0.48
		13–21	7.96	0.53
		22+	7.20	0.45
	Female	0–5	0.00	0.20
		6–12	9.09	0.82
		13–21	3.45	0.24
		22+	11.11	0.56
ADHD	Male	0–5	12.77	0.57
		6–12	8.70	0.66
		13–21	8.85	0.60
		22+	2.40	0.28
	Female	0–5	20.00	1.20
		6–12	27.27	0.82
		13–21	0.00	0.45
		22+	16.67	0.72

Shading indicates highest percent and highest weighted rank score per sex and age group.
